# Individually tailored digital self-care, with and without therapist-guidance

**DOI:** 10.1192/j.eurpsy.2021.73

**Published:** 2021-08-13

**Authors:** M. Kraepelien

**Affiliations:** Clinical Neuroscience, Center For Psychiatry Research, Karolinska Institutet, Stockholm, Sweden

**Keywords:** personalized treatment, Internet, psychological interventions, e-mental health

## Abstract

**Introduction:** Digital mental health services have been a part of routine care at a few locations worldwide since almost 15 years, most often in the form of Internet-based Cognitive Behavioural Therapy (ICBT) with scheduled weekly therapist-guidance. Personalization in the form of individual tailoring of treatment content is promising in ICBT. Digital Self-care, interventions constructed to be self-guided, would need to be constructed carefully to achieve equal levels of adherence and symptom reductions compared to therapist-guided interventions, especially when including individually tailored content. **Objectives:** To construct an individually tailored self-care intervention including a technical solution, acting as a proof of concept that self-guided digital interventions for mental health can be administered in a safe, effective, personalized and cost-effective way. **Methods:** In step I, a new digital platform is created based on the experience from previous successful implementations of ICBT together with experts on user experience. A series of digital mental health tools based on ICBT are tested for safety, usability and credibility. In step II these tools are combined into individually tailored package interventions for different conditions and optimized for greater efficiency. In step III these optimized interventions would be compared to their counterpart therapist-guided interventions in randomized trials. **Results:** Preliminary results from step I will be presented, including the current development of the digital platform and feasibility data from the first three studies.

**Disclosure:**

No significant relationships.

